# Genetic contributions to lupus nephritis in a multi-ethnic cohort of systemic lupus erythematous patients

**DOI:** 10.1371/journal.pone.0199003

**Published:** 2018-06-28

**Authors:** Cristina M. Lanata, Joanne Nititham, Kimberly E. Taylor, Sharon A. Chung, Dara G. Torgerson, Michael F. Seldin, Bernardo A. Pons-Estel, Teresa Tusié-Luna, Betty P. Tsao, Eric F. Morand, Marta E. Alarcón-Riquelme, Lindsey A. Criswell

**Affiliations:** 1 Russell/Engleman Rheumatology Research Center, Department of Medicine, University of California San Francisco, San Francisco, CA, United States of America; 2 Department of Pediatrics, University of California San Francisco, San Francisco, California, United States of America; 3 Department of Biochemistry and Molecular Medicine, University of California Davis, Davis, California, United States of America; 4 Division of Rheumatology and Allergy, Department of Medicine, University of California Davis, Davis, California, United States of America; 5 Hospital Sanatorio Parque, Rosario, Argentina; 6 Instituto Nacional de Ciencias Médicas y Nutrición Salvador Zubiran and Instituto de Investigaciones Biomédicas de la Universidad Nacional Autónoma de Mexico, Mexico City, Mexico; 7 Department of Medicine, Medical University of South Carolina, Charleston, South Carolina, United States of America; 8 School of Clinical Sciences, Monash University Faculty of Medicine, Nursing & Health Sciences, Melbourne, VIC, Australia; 9 Pfizer—University of Granada—Andalusian Government Center for Genomics and Oncological Research (GENYO), PTS, Granada, Spain; 10 Unit for Chronic Inflammatory Diseases, Institute for Environmental Medicine, Karolinska Institute, Solna, Sweden; Peking University First Hospital, CHINA

## Abstract

**Objective:**

African Americans, East Asians, and Hispanics with systemic lupus erythematous (SLE) are more likely to develop lupus nephritis (LN) than are SLE patients of European descent. The etiology of this difference is not clear, and this study was undertaken to investigate how genetic variants might explain this effect.

**Methods:**

In this cross-sectional study, 1244 SLE patients from multiethnic case collections were genotyped for 817,810 single-nucleotide polymorphisms (SNPs) across the genome. Continental genetic ancestry was estimated utilizing the program ADMIXTURE. Gene-based testing and pathway analysis was performed within each ethnic group and meta-analyzed across ethnicities. We also performed candidate SNP association tests with SNPs previously established as risk alleles for SLE, LN, and chronic kidney disease (CKD). Association testing and logistic regression models were performed with LN as the outcome, adjusted for continental ancestries, sex, disease duration, and age.

**Results:**

We studied 255 North European, 263 South European, 238 Hispanic, 224 African American and 264 East Asian SLE patients, of whom 606 had LN (48.7%). In genome-wide gene-based and candidate SNP analyses, we found distinct genes, pathways and established risk SNPs associated with LN for each ethnic group. Gene-based analyses showed significant associations between variation in *ZNF546* (p = 1.0E-06), *TRIM15* (p = 1.0E-06), and *TRIMI0* (p = 1.0E-06) and LN among South Europeans, and *TTC34* (p = 8.0E-06) was significantly associated with LN among Hispanics. The SNP rs8091180 in *NFATC1* was associated with LN (OR 1.43, p = 3.3E-04) in the candidate SNP meta-analysis with the highest OR among African-Americans (OR 2.17, p = 0.0035).

**Conclusion:**

Distinct genetic factors are associated with the risk of LN in SLE patients of different ethnicities. CKD risk alleles may play a role in the development of LN in addition to SLE-associated risk variants. These findings may further explain the clinical heterogeneity of LN risk and response to therapy observed between different ethnic groups.

## Introduction

Lupus nephritis (LN) is a severe manifestation of systemic lupus erythematosus (SLE) affecting approximately 40–70% of patients, and contributes substantially to the overall morbidity and mortality of this disease [1]. Hispanic, African American, and Asian patients develop SLE at a younger age and more severe manifestations including LN, than patients of European descent [2]. The etiology of these ethnic disparities is a matter of ongoing debate, with genetic and non-genetic factors being implicated [3]. Previous work has shown that European ancestry is protective for LN whereas Amerindian and African ancestry contributes to risk [4, 5]. While establishing that genetic ancestry is important, studies seeking to define the genetic risk factors driving LN among SLE patients of different ethnicities have been inconclusive [4]. This might be due to several factors, such as the risk loci examined were associated with SLE but might not be relevant to the risk of LN, or the platforms used did not have adequate genome-wide coverage for the studied ethnic group. Finally, SLE-associated risk loci have mainly been described in populations of European ancestry, although combined studies of European and Chinese SLE suggest that the majority of SLE susceptibility loci overlap between these distinct population groups [6]. The current study aims to better define genetic variants that could explain the differential risk of LN across ethnic groups.

Genetic variants other than SLE-associated risk alleles may be relevant to the ethnic-specific risk of LN. Murine models of SLE and LN have indicated that interactions between genes with different functions contribute to the development of severe LN. These genes include not only those involved in immune processes, but also genes governing renal function or the handling of apoptotic debris [1]. Supporting this view, a genome-wide association study (GWAS) of LN in SLE patients of European descent revealed that the strongest association fell outside of the MHC region, at a variant located close to *PDGFRA*, which had not been identified in SLE risk GWAS [7]. Furthermore a variant in *APOL1* has been implicated in the development of early onset of kidney failure as well as end-stage renal disease in African Americans. This risk variant has also been associated with LN in patients of African American descent [8]. To better examine how genetic risk factors for LN may differ between difference ethnic groups, we performed genome-wide SNP genotyping on a well-characterized cohort of lupus patients from 5 ethnic groups: North European, South European, Asian, Hispanic, and African American. Individuals of European ancestry were divided into North and South European groups as this sub-stratification confers distinct risk for particular SLE phenotypes [9, 10]. Our approach included gene-based association testing utilizing genome-wide data as well as a candidate SNP analysis of established risk variants for SLE, LN and chronic kidney disease (CKD). We observed that the genes and pathways most associated with LN differ across ethnicities. Genes not known to be associated with LN, such as *TRIM10*, *TRIM15*, and *ZNF456* were associated with LN only among South Europeans, and *TTC34* was associated with LN among Hispanics. Furthermore, our candidate SNP analysis revealed several chronic kidney disease (CKD) risk SNPs associated with LN. Distinct genetic risk factors may influence the risk of LN among ethnicities, which could contribute to the difference in prevalence of LN different ethnic groups.

## Materials and methods

### Participants

#### Ethics statement

Written informed consent was obtained from all study participants and the institutional review board at each collaborating center approved the study (institutional review board of the University of San Francisco California, institutional review board of Oklahoma Medical Research Foundation, Monash Health Human Research Ethics Committee and the institutional review board of the University of California Los Angeles).

We studied East Asian, Hispanic, North European, South European, and African American patients from established lupus cohorts. A total of 1273 SLE cases were obtained from the US (n = 888), Australia (n = 76), Spain (n = 160), and Mexico (n = 120). All participants fulfilled the American College of Rheumatology (ACR) revised classification criteria for SLE [11]. Participants were grouped according to self-reported ethnicity. All research was approved by an institutional review board or appropriate ethics committee at each site. Participants were recruited from a variety of settings, including academic medical centers and community hospitals. Table 1 shows the distribution of these participants by self-reported ethnicity. Our primary outcome variable was the presence of LN, defined as fulfilling the ACR classification criteria for renal manifestation of SLE (>0.5 grams of proteinuria per day or 3+ protein on urine dipstick analysis) or having evidence of LN on kidney biopsy.

**Table 1:** Clinical Characteristics of the 1,244 participants with systemic lupus erythematosus.

Characteristics*	North European n = 255	South European n = 263	Asian n = 238	African American n = 224	Hispanic n = 264
Female n (%)	224 (87.9)	238 (90.2)	213 (89.5)	212 (94.6)	245 (93.2)
Age of Onset (mean in years, +/- SD)	36 +/- 14	31 +/- 12	28 +/- 12	33 +/- 13	30 +/- 11
SLE Duration (mean in years, +/- SD)	9.2 +/- 8	10.6 +/- 9	8.3 +/- 8	8.8 +/- 8	7.1 +/- 7
Lupus Nephritis n (%)	94 (37)	106 (40.5)	146 (61.9)	123 (55.2)	137 (52.1)
Anti dsDNA positive n (%)	130 (50.1)	104 (62.3)	179 (75.2)	148 (66.1)	149 (67.1)

*SD = standard Deviation

### Genotyping, imputation and quality assurance

DNA was collected from blood or saliva (Oragene DNA sample collection kits, DNAGenotek) from all study participants. All participants were genotyped simultaneously using the Affymetrix LAT1 World array at the University of California, San Francisco, Institute of Human Genetics Genomics Core Facility. This high-throughput genotyping array is composed of 817,810 single nucleotide polymorphism (SNP) markers across the genome and was specifically designed to maximize coverage for diverse ethnic populations, including West Africans, Europeans and Native Americans [12]. Samples were filtered based on having call rate < 95%, discrepancies between reported and genetic assessed gender, and evidence of relatedness (one of each first degree relative pairs removed, defined by identity by descent pi-hat > 0.25). For evidence of departure from Hardy-Weinberg equilibrium (HWE), we chose to examine LN negative and dsDNA negative participants among the North European and Asian ethnic groups. These ethnic groups were the most homogeneous with regards to continental genetic ancestry. SNPs were removed from analysis if evidence of departure from HWE was present (p < 5 E-08 in self-identified Europeans and p < 1 E-05 in self-identified Asians) and if genotyping call rates were below 95%. Standard Affymetrix Axiom metrics were also applied (DQC ≥0.82 and default cluster metrics of SNPolisher). After applying these quality control assessments, 1244 participants and 801,067 SNPs remained in analysis. Genotypes of variants that passed quality control underwent imputation on the University of Michigan Imputation Server (imputationserver.sph.umich.edu/index.html). Imputation was performed using the 1000 genomes phase 3 reference panels for each ethnic group. Variants with imputation quality *INFO score <0*.*7* and minor allele frequency <1% were excluded from the analysis [13].

### Statistical analysis

#### Ancestry calculations

Because self-identified ethnicity is an imprecise proxy for the actual genetic ancestry of an individual, we used SNP data to estimate the overall genetic ancestry (i.e., % ancestry of the 5 ethnic groups under study) for each participant using the program ADMIXTURE[14] (S1 Fig). We ran a supervised calculation assuming admixture with 5 parental populations, utilizing available data from the HapMap project (TSI and Basque for South Europeans, CEU, AFR, EAS, and PIMA) [15], the Human Genome Diversity Project [16], and collaborators (M. Seldin, University of California, Davis). From a set of established ancestry informative markers, 609 autosomal SNPs genotyped on the Affymetrix LAT1 World array that overlapped with all the reference panels were used to estimate genetic ancestry.

#### Gene-based tests of association

Initial analyses included gene-based tests of association with LN. These methods can identify the enrichment of multiple SNPs associated with the disease/trait when the association for individual SNPs may not reach genome-wide significance [17]. Genome wide-association testing was done within each ethnic group using logistic regression modeling with LN status as the outcome, and each SNP as the primary predictor, adjusting for sex, age, disease duration, and genetic ancestry utilizing PLINK version 1.90 beta [18]. Gene-based tests of association were performed separately for each ethnic group utilizing all genes with SNPs that passed quality assurance measures (i.e., a genome-wide assessment). VEGAS2 [19] was used to assign SNPs to genes (within 50KB, using Human Genome version 19) and produce gene-based test statistics and empirical p values by simulation. Varying patterns of LD were taken into consideration in the gene-based analysis by selecting the most relevant reference population. Pathway analysis under this approach consists of aggregating association strength of individual markers into pre-specified biological pathways. This method accounts for gene size and linkage disequilibrium between markers using simulations from the multivariate normal distribution. Pathway size is taken into account via a resampling approach [20].

#### Ethnic-specific candidate SNP analysis

After review of large published GWAS, association studies, and meta-analyses, 202 SNPs with replicated associations with SLE, LN and/or CKD were selected for analysis (S1–S3 Tables). Among these 202 SNPs, 67 were genotyped and 137 were imputed. Association testing was done within each ethnic group using logistic regression modeling with LN status as the outcome, and each SNP as the primary predictor, adjusting for sex, age, disease duration, and genetic ancestry utilizing PLINK version 1.90 beta [18]. Association summary statistics for each candidate gene SNP were combined using meta-analysis techniques across ethnic groups in PLINK under a random effects model. Heterogeneity in allelic effects between ethnic groups at each variant was assessed using Cochran’s Q and the I-square statistics [21].

## Results

Of the 1244 SLE participants, 606 patients had LN (48.7%). This varied significantly according to ethnicity (Table 1), with the highest rate of LN observed in Asians (61%) and the lowest rate observed in North Europeans (37%). Genetic admixture was seen in multiple self-identified ethnic groups and in particular the Hispanic population, where the mean Native American (NA) ancestry was 46% (S1 Fig). Both North and South European populations had significant intercontinental admixture between Northern and Southern Europe. The African American population in this study had a small degree of admixture with 8% mean of North European ancestry; however, some African American individuals had up to 60% North European ancestry.

As reported previously, we observed a protective effect of European ancestry with LN across the 5 ethnicities adjusting for age, sex, and disease duration (Table 2). Although East Asian and African ancestry were associated with LN, after adjusting for other ancestries, only the European protective effect (North and South) persisted.

**Table 2:** Association between continental ancestry and lupus nephritis among 1244 SLE patients.

Model **	OR (95% CI) for LN given 25% increase in ancestry*	P
Model 1 (NE ancestry, disease duration, sex)	0.84 (0.77–0.92)	0.00016
Model 2 (SE ancestry, disease duration, sex)	0.79 (0.71–0.87)	4.94 E-06
Model 3 (NA ancestry, disease duration, sex)	1.05 (0.92–1.21)	0.465
Model 4 (EAS ancestry, disease duration, sex)	1.22 (1.12–1.32)	6.11 E-06
Model 5 (AFR ancestry, disease duration, sex)	1.14 (1.04–1.25)	0.005
Model 6:		
NE + other genetic ancestries, disease duration, sex	0.82 (0.7–0.96)	0.013
SE + other genetic ancestries, disease duration, sex	0.77 (0.65–0.91)	0.003
EAS + other genetic ancestries, disease duration, sex	1.05 (0.86–1.18)	0.48
AFR + other genetic ancestries, disease duration, sex	1.01 (0.86–1.27)	0.85

* OR = odds ratio, 95% CI = 95% Confidence Interval

** Adjusted values between parentheses. NE = North European, SE = South European, NA = Native American, EAS = Asian, AFR = African, LN = lupus nephritis

Using gene-based tests of associations within each ethnic group, 4 genes were significantly associated with LN (p<1.0E-06, Bonferroni threshold, alpha = 2.5x10^-6^ for 20,000 gene tests) in 2 ethnic groups (Table 3). Four of the top 5 gene-based associations within each racial/ethnic group had p<0.05 in at least one other racial/ethnic group (*SYNJ2*, *MMEL1*, *FAM213B*, and *OR10C1*). *TRIM10* and *TRIM15* encode for 2 members of the superfamily of tripartite motif-containing (TRIM) proteins. *ZNF546* encodes for a transcription factor of the ZNF family that has not been associated with autoimmunity or kidney disease. Among the strongest 10 associations with LN among South Europeans, there was enrichment for TRIM protein coding genes (p = 2.1E-10) such as *TRIM26* (p = 2.2E-05), *TRIM31* (p = 4E-05) and *TRIM40* (p = 6E-05) (S4 Table). Although these genes are at the MHC-I region, the top SNP within TRIM10 and TRIM15 in the gene based analysis harbors no linkage disequilibrium between SLE associated SNPs in the HLA-DR region. *TTC43*, associated in Hispanics, encodes for an uncharacterized protein that has recently been implicated by computational approaches as a potential auto-antigen in SLE [22].

**Table 3:** Genes with the strongest evidence of association with lupus nephritis within each ethnic group.

		NE	SE	HI	AA	AS	Meta*
CHR	gene	P	P	P	P	P	P
*North European*							
19	*AURKC*	6.9E-05	0.66	0.59	0.42	0.80	0.36
19	*ZNF805*	9.4E-05	0.76	0.52	0.44	0.86	0.22
19	*ZNF264*	9.9E-05	0.68	0.61	0.63	0.97	0.27
19	*ZNF460*	0.00016	0.80	0.46	0.63	0.55	0.13
5	*SLC36A1*	0.00034	0.64	0.82	0.55	0.27	0.07
*South European*							
6	***TRIM15***	0.17	**1.0E-06**	0.44	0.92	0.35	0.93
6	***TRIM10***	0.18	**1.0E-06**	0.41	0.87	0.41	0.80
19	***ZNF546***	0.93	**1.0E-06**	0.67	0.14	0.75	0.61
19	*ZNF780B*	0.86	1.2E-05	0.66	0.23	0.64	0.39
6	*TRIM26*	0.15	2.2E-05	0.43	0.90	0.51	0.001
*Hispanic*							
1	***TTC34***	0.48	0.173	**8E-06**	0.55	0.07	0.49
6	*NRM*	0.55	0.548	1.2E-05	0.65	0.60	0.002
1	*MMEL1*	0.72	0.037	3E-05	0.58	0.07	0.64
6	*PPP1R18*	0.55	0.492	3.7E-05	0.68	0.54	0.005
1	*FAM213B*	0.68	0.037	4.3E-05	0.57	0.06	0.001
*African American*							
6	*SYNJ2*	0.01	0.490	0.36	2.1E-05	0.43	0.60
6	*SERAC1*	0.30	0.753	0.62	2.7E-05	0.67	0.92
17	*UNC13D*	0.07	0.134	0.81	0.00010	0.22	0.93
6	*OR2H1*	0.23	0.080	0.28	0.00013	0.55	0.009
6	*OR10C1*	0.28	0.006	0.22	0.00016	0.59	0.001
*Asian*							
8	*NSMCE2*	0.47	0.90	0.44	0.21	0.00022	0.86
10	*FANK1*	0.74	0.37	0.47	0.63	0.00057	0.95
2	*ICOS*	0.84	0.62	0.19	0.69	0.00063	0.66
1	*FAM151A*	0.39	0.01	0.78	0.21	0.00064	0.29
20	*DEFB126*	0.38	0.64	0.10	0.40	0.00094	0.67
*Meta-Analysis*							
18	*LOC400644*	0.13	0.09	0.20	0.31	0.03	0.0002
2	*LOC101929753*	0.006	0.19	0.10	0.73	0.56	0.0003
13	*SLC25A15*	0.06	0.19	0.01	0.14	0.11	0.0007
22	*TUBA3FP*	0.07	0.61	0.58	0.61	0.81	0.0012
3	*SPICE1*	0.46	0.65	0.47	0.07	0.09	0.0022

*NE = North European SE = South European HI = Hispanic AA = African American AS = Asian Meta = Meta-analysis

Pathway analyses utilizing genome-wide data did not reveal statistically significant results (p < 1 E-05)[19]. The most associated pathways for each ethnic group were different; these pathway analyses identified top associations at dysregulated immune signaling and fibrotic responses in the pathogenesis of LN (Table 4). The top network in North Europeans was receptor-regulated SMAD binding (p = 0.0002). SMADs activate transcription of TGF-β target genes. In Hispanics, the top network implicated was the CD28 dependent Vav1 signaling pathway (p = 0.0007). The second top network in Hispanics was related to 1L-12 mediated signaling events (p = 0.0014). In Asians, the top networks included the p38MAPK pathway, (p = 0.0007), the mTOR signaling pathway (p = 0.0022) and the family of inhibitor of differentiation (ID) proteins (p = 0.0023). Finally, in the meta-analysis, TNF-related pathways showed the strongest evidence of association.

**Table 4:** Pathways with the strongest evidence of association with lupus nephritis within each ethnic group.

		Pathway	NE	SE	HI	AA	AS	Meta*
	Accession		P	P	P	P	P	P
*North European*								
	M13070	R-SMAD binding	0.0002	0.62	0.30	0.78	0.92	0.15
	M562	Facilitative Na+-independent glucose transporters	0.0004	0.08	0.84	0.38	0.67	0.67
	M1012	Hexose transmembrane transporter activity	0.0008	0.36	0.67	0.14	0.08	0.47
	GO:0005355	Glucose transmembrane transporter activity	0.0009	0.26	0.69	0.15	0.09	0.40
	GO:0031056	Regulation of histone modification	0.0012	0.66	0.60	0.24	0.27	0.12
*South European*								
	GO:0070412	Hormone ligand-binding receptors	0.61	0.0003	0.54	0.73	0.05	0.02
	M8472	GRB2 events in ERBB2 signaling	0.72	0.001	0.93	0.63	0.79	0.40
	GO:0015149	Interleukin-2 signaling	0.92	0.001	0.52	0.74	0.96	0.72
	M1718	IL4 receptor signaling in B lymphocytes	0.54	0.002	0.81	0.79	0.84	0.50
	RHSA74751	Insulin receptor signaling cascade	0.97	0.002	0.96	0.56	0.38	0.19
*Hispanic*								
	M13618	CD28 dependent Vav1 pathway	0.19	0.29	0.0007	0.80	0.24	0.08
	PID M54	IL12-mediated signaling events	0.49	0.35	0.0014	0.35	0.73	0.35
	PID M84	ATM pathway	0.61	0.71	0.0014	0.97	0.95	0.48
	RHSA428540	Activation of RAC1	0.23	0.42	0.0014	0.89	0.25	0.32
	GO:0016254	Preassembly of GPI anchor in ER membrane	0.37	0.72	0.0019	0.84	0.28	0.97
*African American*								
	GO:0003677	DNA binding	0.44	0.81	0.74	0.0024	0.35	0.46
	P02772	Pyruvate metabolism	0.85	0.06	0.92	0.0046	0.14	0.28
	GO:0000315	Organellar large ribosomal subunit	0.85	0.06	0.92	0.0046	0.14	0.28
	M5940	Endocytotic role of NDK, phosphins and dynamin	0.17	0.81	0.83	0.0047	0.47	0.35
	GO:0006109	Regulation of carbohydrate metabolic process	0.75	0.92	0.25	0.0071	0.76	0.82
*Asian*								
	M76	p38 signaling mediated by MAPKAP kinases	0.92	0.57	0.40	0.87	0.0007	0.10
	M16563	mTOR signaling pathway	0.74	0.28	0.99	0.42	0.0022	0.33
	NetPath	ID signaling pathway	0.05	0.84	0.45	0.63	0.0043	0.27
	M229	Signaling mediated by P38-alpha and P38-beta	0.82	0.14	0.43	0.71	0.0023	0.08
	GO:0008191	Metalloendopeptidase inhibitor activity	0.87	0.85	0.30	0.51	0.0055	0.92
*Meta-analysis*								
	GO:0005031	TNF-activated receptor activity	0.85	0.04	0.003	0.47	0.31	0.0027
	GO:0005035	Death receptor activity	0.82	0.05	0.007	0.62	0.41	0.0040
	GO:0043120	TNF binding	0.87	0.05	0.005	0.51	0.41	0.0046
	GO:0005072	TGF B receptor cytoplasmic mediator activity	0.03	0.07	0.56	0.31	0.96	0.0061
	GO:0005071	Serine/threonine kinase signaling protein activity	0.05	0.07	0.66	0.24	0.95	0.0091

*NE = North European SE = South European HI = Hispanic AA = African American AS = Asian Meta = Meta-analysis

### Candidate SNP analysis

Table 5 shows the most significantly associated SNPs within each ethnicity in comparison to the other ethnic groups. The associations between candidate SNPs and LN did not reach statistical significance in any specific ethnic group. However, the most significantly associated SNPs for each ethnic group are largely distinct, with only one SNP overlapping between 2 ethnic groups (rs3184504 in *SH2B3* for both North and South Europeans). Of note, many of these top SNPs are risk alleles for CKD and not SLE, such as *ETV4* and *GCKR* in Hispanics, *NFATC1* and *SYPL2* in African Americans, *DAB2* and *KBTBD2* in Asians, and *PRKAG2* in South Europeans.

**Table 5:** Candidate SNP analysis with the strongest evidence of association with lupus nephritis within each ethnic group.

				North European			South European			Hispanic			African American			Asian			Meta-Analysis*		
CHR	SNP ID	gene	RA	RAF	OR	P	RAF	OR	P	RAF	OR	P	RAF	OR	P	RAF	OR	P	OR	P	I^2^%
*North European*																					
12	rs17696736	*TRAFD1*	G	0.48	1.68	**0.009**	0.49	1.63	0.0085	0.25	1.14	0.53	0.08	0.52	0.09	0.01	1.21	0.88	1.26	0.22	**56**
10	rs1913517	*WDFY4*	A	0.56	1.64	**0.016**	0.49	1.25	0.23	0.54	0.68	0.04	0.36	1.04	0.88	0.35	1.40	0.11	1.15	0.39	**67**
6	rs2327832	*TNFAIP3*	G	0.27	0.61	**0.036**	0.20	0.97	0.90	0.09	1.57	0.17	0.11	1.99	0.05	0.01	1.96	0.59	1.13	0.62	**62**
6	rs6920220	*TNFAIP3*	A	0.27	0.61	**0.036**	0.20	0.97	0.88	0.10	1.58	0.15	0.12	2.19	0.03	0.01	1.96	0.59	1.16	0.55	**66**
12	rs3184504	*SH2B3*	T	0.53	1.52	**0.037**	0.53	1.69	**0.0058**	0.26	1.06	0.76	0.09	0.7	0.33	0.01	1.69	0.67	1.28	0.09	37
*South European*																					
14	rs8012283	*NIN*, *SAV1*	G	0.18	1.43	0.15	0.19	2.25	**0.0016**	0.10	0.70	0.25	0.41	0.81	0.30	0.02	0.70	0.65	1.12	0.63	**70**
11	rs2732552	*CD44/PDHX*	C	0.60	1.08	0.71	0.59	0.56	**0.004**	0.69	0.88	0.54	0.40	1.39	0.14	0.80	0.70	0.18	0.88	0.42	**64**
7	rs10254284	*JAZF1*	A	0.36	1.47	0.06	0.39	1.81	**0.0045**	0.59	1.49	0.03	0.42	0.79	0.22	0.95	2.04	0.22	1.37	0.07	**62**
7	rs7805747	*PRKAG2*	A	0.31	1.51	0.04	0.28	0.55	**0.0046**	0.18	0.98	0.95	0.29	1.05	0.83	0.01	2.57	0.51	0.98	0.93	**68**
*Hispanic*																					
7	rs1635852	*JAZF1*	T	0.53	2.43	0.41	0.55	1.43	0.06	0.64	1.81	**0.003**	0.73	1.04	0.87	0.79	0.82	0.41	1.22	0.10	47
7	rs849142	*JAZF1*	T	0.52	1.15	0.47	0.55	1.39	0.08	0.62	1.75	**0.004**	0.79	0.84	0.49	0.97	0.8	0.73	1.25	0.09	37
3	rs10513801	*ETV5*	G	0.12	1.01	0.96	0.11	0.86	0.60	0.05	3.66	**0.009**	0.01	0.75	0.78	0.06	1.45	0.39	1.27	0.35	43
2	rs1260326	*GCKR*	T	0.38	1.20	0.36	0.44	0.89	0.52	0.33	0.6	**0.013**	0.14	0.76	0.34	0.46	1.04	0.85	0.88	0.32	41
1	rs17484292	*NMNAT2*	T	0.91	1.04	0.90	0.9	1.04	0.90	0.94	0.32	**0.021**	0.98	1.11	0.91	0.00	0.00	NA	0.81	0.45	38
*African American*																					
**18**	**rs8091180**	***NFATC1***	**A**	**0.61**	**1.14**	**0.56**	**0.58**	**1.39**	**0.09**	**0.68**	**1.48**	**0.04**	**0.18**	**2.17**	**0.00357**	**0.80**	**1.27**	**0.36**	**1.43**	**0.00033**	**0**
1	rs1050501	*FCGR2B*	C	0.12	0.54	0.08	0.14	1.38	0.21	0.08	0.96	0.91	0.24	0.49	**0.00497**	0.30	0.65	0.08	0.75	0.15	**61**
6	rs6568431	*ATG5/PRDM1*	A	0.42	1.47	0.68	0.4	1.02	0.90	0.32	0.98	0.93	0.43	0.57	**0.00845**	0.43	1.08	0.72	0.92	0.53	40
1	rs12136063	*SYPL2*	A	0.71	0.72	0.13	0.75	0.92	0.72	0.86	0.57	0.04	0.34	1.79	**0.00958**	0.93	1.03	0.95	0.93	0.74	**69**
1	rs2022013	*NMNAT2*	C	0.4	1.28	0.20	0.38	0.96	0.82	0.35	1.42	0.05	0.67	1.78	**0.0111**	0.34	1.26	0.30	1.29	0.0079	15
*Asian*																					
5	rs11959928	*DAB2*	A	0.39	1.06	0.77	0.50	1.29	0.15	0.34	1.24	0.29	0.29	1.07	0.74	0.19	0.40	**0.0014**	0.98	0.91	**70**
2	rs1990760	*IFIH1*	T	0.66	1.44	0.10	0.61	1.05	0.79	0.47	1.18	0.35	0.17	0.82	0.44	0.26	1.93	**0.01**	1.22	0.11	41
7	rs3750082	*KBTBD2*	A	0.38	1.34	0.14	0.37	1.25	0.23	0.25	0.90	0.63	0.60	1.30	0.19	0.36	1.84	**0.011**	1.28	0.01	18
7	rs34350562	*IRF5_TNPO3*	G	0.20	0.95	0.84	0.18	0.86	0.54	0.29	1.32	0.17	0.06	0.94	0.89	0.03	0.09	**0.016**	0.94	0.74	**51**
1	rs17301013	*RABGAP1L*	T	0.48	1.24	0.27	0.46	1.08	0.69	0.38	0.82	0.25	0.82	1.20	0.50	0.58	0.61	**0.019**	0.95	0.68	**51**

* RA = Risk Alelle RAF = risk allele frequency, OR = odds ratio

**Fig 1:**
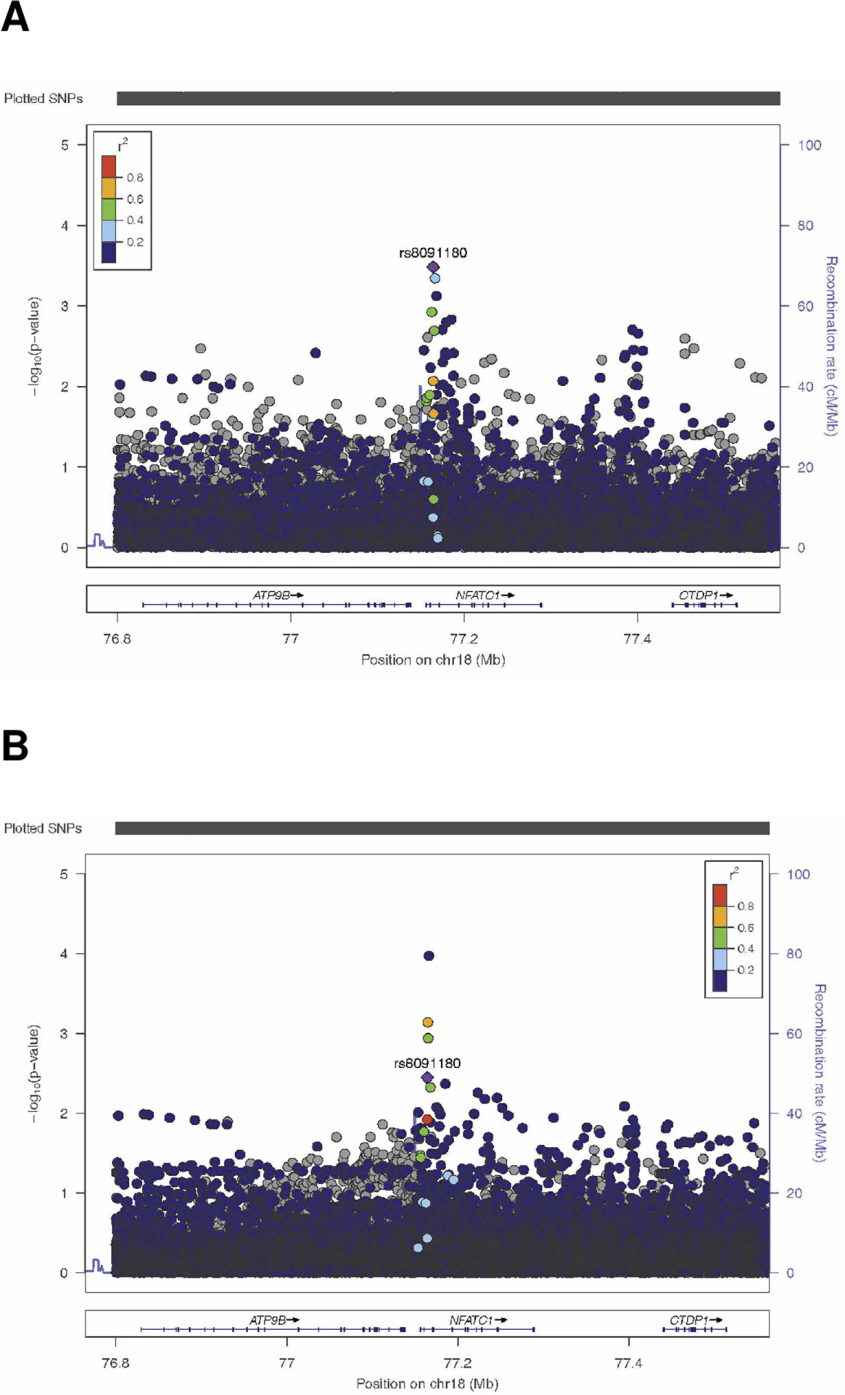
Locus plots of association of SNPs in the *NFATC1* region with renal disease. (A) All SLE participants. (B) African-American participants.

To investigate whether different genetic variants contribute to LN susceptibility across different ethnic groups, we constructed a genetic risk score (GRS), comprised of candidate SNPs with p<0.05 for association with LN. The GRS was calculated for each individual by summing number of risk alleles for each SNP in the GRS. The GRS was not predictive of LN when looking across all individuals, or when looking within ethnic groups (data not shown). We then constructed a ethnic-specific GRS, comprised of candidate SNPs with p<0.05 for association with LN in that ethnic group. The distribution of the 5 ethnic-specific GRS was significantly different across groups (ANOVA, p <0.0007), which supports the hypothesis. Only the GRS calculated from the top North European SNPs was a significant predictor of LN (p = 0.0001 in NE, p = 0.03 in other populations combined) (S5 Table). In the candidate SNP analysis, rs8091180 in *NFATC1* was most highly associated with LN across ethnic groups (trans-ethnic meta-analysis OR 1.43, p = 0.0003). The effect was stronger in the African Americans, with an OR of 2.17 (p = 0.00357). We utilized the genome-wide data available to examine the region. When looking at the entire cohort, this SNP remained the strongest signal in the region (Fig 1A). However, when examining the region among African Americans, the imputed SNP rs68734 appeared to be more strongly associated (p-value = 1.06E−4) (Fig 1B).

As observed in the meta-analysis (Table 5), 13 out of 25 of the most significantly associated SNPs had an I^2^ of greater that 50% suggesting heterogeneity. We examined whether the association of these previously established risk alleles was different among the ethnic groups. Several SNPs had a p-value for the Q statistic approaching statistical significance of 0.0005 after adjusting for multiple comparisons. The most extreme differences between population pairs were between Asians and South Europeans for rs11959928 in *DAB2* (Q = 0.0005, I^2^ = 91.63) and between North and South Europeans for rs7805747 in *PRKAG2* (Q = 0.0006, I^2^ of 91.5). In both scenarios, the effect estimates of the minor alleles were in different directions (risk versus protective).

## Discussion

This study aimed to identify genetic variants contributing to the differential risk of LN among SLE patients of different ethnicities. Overall, in gene-based, pathway, and candidate SNP analyses, we found that the genetic associations differed between ethnic groups. We found novel associations with 4 genes as well as distinct biological pathways associated with LN in particular ethnic groups. We also found evidence of a potential association of SNP rs8091180 in the *NFATC1* gene with lupus nephritis, which was strongest in for African-Americans and has not been previously associated with LN or SLE. Finally, the results of our study highlight the importance of CKD risk loci over SLE risk loci for the development of nephritis in SLE.

Using a genome-wide gene-based approach, we saw an enrichment of members of the TRIM family genes associated with LN only among South Europeans. The TRIM proteins have important roles in innate immunity and antiviral response, in particular in retroviral restriction and antiviral defense [23]. This is consistent with the well-established interferon (IFN) response in SLE, as well as newer evidence implicating activation of retroviral elements as potential triggers of SLE [24, 25]. The function of TRIM10 remains unknown. TRIM15 has been found to be up-regulated in human THP1-derived macrophages after activation with TLR3 ligands [26]. TLR3 recognizes double-stranded RNA (the genetic basis of retroviruses) and upon recognition, it induces the activation of IRF3 to increase production of type I IFNs. This is suggestive of linking a potential variant influencing the function of at least TRIM15, which would have an effect on the ability to restrict endogenous retro-elements from inducing an IFN response. If confirmed in independent datasets, additional work will be required to understand why these genes are associated with LN only among South Europeans, although differences in selection pressures in different geographic regions might explain such differences [27]. Due to the nature of the gene-based analysis, we do not have the direction of the association (protective or risk), but we know that Europeans are protected from LN in comparison to other populations [4].

In Hispanics, *TTC34* was associated with LN. This gene is significantly enriched with simple tandem repeat (STR) sequences, conferring vulnerability to somatic mutation. It was recently described as a potential autoantigen, in particular in SLE [28]. We are not aware of previous reports of anti-TTC34 antibodies in serum of SLE patients.

LN continues to be a therapeutic challenge, and there continues to be a great need for more effective treatments. Even with advances in care, the 10-year cumulative incidence of ESRD is 10.1% and of death is 5.9% [29]. Understanding the complex heterogeneity of LN is key to developing more targeted treatments. Treatment response in LN also differs between ethnicities [29]. Although these findings will need to be replicated in other cohorts, it is of interest that the pathways with the strongest evidence of association were biologically relevant to renal disease and differed across ethnicities. These findings could inform future treatment strategies. For example, in Asians, one of the top pathways included the mTOR signaling pathway, which has been implicated in both SLE and LN pathogenesis [30, 31]. Another interesting pathway in this particular group is the family of inhibitor of differentiation (ID) proteins, which have been shown to play a role in steering multipotent progenitors away from the lymphoid lineages, thus allowing them to differentiate into myeloid and dendritic cells [32]. Targeting the SMAD/TGF pathway, the top pathway associated in North Europeans, might be an effective strategy for this group. TGFβ-SMAD signaling has a central role in kidney fibrosis and progression to CKD, which has led to SMADs being recently identified as therapeutic targets for CKD [33, 34].

When examining previously established candidate risk SNPs, we observed the same pattern: the SNPs with the strongest evidence of association differed in each ethnic group. In addition, there was an association of SNP rs809180 in *NFATC1* with LN across ethnicities. *NFATC1* is a validated CKD risk loci and encodes for nuclear factor of activated T-cells (calcineurin dependent 1) that is involved in the activation of the T-cell antigen receptor [35, 36]. We sought to replicate this finding in an independent cohort of 1620 African American lupus patients, as we observed the strongest association with LN among this group. Sixty-six SNPs in the *NFATC1* region were examined for association with lupus nephritis. Although rs8091108 did not replicate, [OR 1.02 95%CI 0.85–1.23, p = 0.8] two other SNPs within this gene where associated with LN, rs11660906 (OR 0.45 95% CI 0.27–0.74, p = 0.0013,) and rs11663132 (OR 0.46 95%CI 0.28–0.76, p = 0.0018), indicating that variants in this region may be associated with LN. *NFATC1* is targeted by cyclosporine and tacrolimus, which have been used to prevent renal transplant rejection and treat some cases of LN [37]. Furthermore, *NFATC1* has been recently described as an important regulator of cytotoxic T lymphocyte effector functions [35]. Experimental studies showed that a loss-of-function mutation in the ancestral orthologue of NFATC1 in *Drosophila melanogaster* was associated with altered sensitivity to salt stress, suggesting a role for this gene in ionic or osmotic regulation [38]. Endogenous signals, such as interstitial osmolality, may influence the immune kidney landscape. Increased extracellular sodium skews CD4 T cells to a Th17 phenotype [39, 40] and kidney medullary hypersalinity has shown to cause NFAT5-dependent recruitment of circulating monocyte-derived mononuclear phagocytes into the region [41]. Therefore, a high salt intake coupled with a genetic predisposition to salt sensitivity might contribute to renal disease in SLE patients.

A major strength of our study is the incorporation of multiple ethnic groups and their assessment using the same genotyping platform. This design allowed for the simultaneous examination of predisposing loci for renal disease among all study participants. Our candidate SNP analysis incorporated risk loci not only for SLE, but also LN and CKD. A recognized limitation of our study is the relatively small sample size within each ethnic group, which limited our statistical power for ethnic-specific analyses. Another limitation is that our candidate SNP study was limited to SNPs that primarily have been identified and validated in populations of European descent. In support of this bias, the GRS was only significantly associated with LN in North Europeans. Therefore, we cannot exclude the presence of one or more potentially functional variants (common or rare) in loci not identified in the current study.

## Conclusions

In summary, we provide evidence of ethnic-specific genetic factors influencing the risk of LN among SLE patients, with corroboration of our findings (e.g., the association of *TRIM10* and *TRIM15* in South Europeans) needed in future studies. While fine mapping is needed to pinpoint causal variation in relevant populations, this study represents progress in elucidating the genetic underpinnings driving LN among SLE patients of different ethnicities.

## Supporting information

S1 Fig Genetic ancestry according to ethnicity. Admixture analysis of 1244 patients with systemic lupus erythematosus from five different ethnicities.
(TIF)

S1 Table Association of previously identified loci with estimated glomerular filtration rate (eGFR). (DOCX)

S2 Table Association of previously identified loci with lupus nephritis. (DOCX)

S3 Table Association of previously identified loci with systemic lupus erythematosus. (DOCX)

S4 Table Genes with the strongest evidence of association with lupus nephritis among South European systemic lupus erythematosus patients. (DOCX)

S5 Table Associations between lupus nephritis and ethnic-specific genetic risk scores (GRS). (DOCX)

## References

[1] Mohan C, Putterman C. Genetics and pathogenesis of systemic lupus erythematosus and lupus nephritis. Nature Reviews Nephrology. 2015;11(6):329–41. doi: 10.1038/nrneph.2015.33 25825084 10.1038/nrneph.2015.33

[2] Feldman CH, Hiraki LT, Liu J, Fischer MA, Solomon DH, Alarcõn GS, et al Epidemiology and sociodemographics of systemic lupus erythematosus and lupus nephritis among US adults with Medicaid coverage, 2000–2004. Arthritis and Rheumatism. 2013;65(3):753–63. doi: 10.1002/art.37795 23203603 10.1002/art.37795 PMC3733212

[3] Tesar V, Hruskova Z. Lupus Nephritis: A Different Disease in European Patients? Kidney Dis (Basel). 2015;1(2):110–8. 27536671 10.1159/000438844 PMC4934820

[4] Richman IB, Taylor KE, Chung SA, Trupin L, Petri M, Yelin E, et al European genetic ancestry is associated with a decreased risk of lupus nephritis. Arthritis & Rheumatism. 2012;64(10):3374–82. 23023776 10.1002/art.34567 PMC3865923

[5] Richman IB, Chung SA, Taylor KE, Kosoy R, Tian C, Ortmann WA, et al European population substructure correlates with systemic lupus erythematosus endophenotypes in North Americans of European descent. Genes and immunity. 2010;11(6):515–21. doi: 10.1038/gene.2009.80 19847193 10.1038/gene.2009.80 PMC3951966

[6] Morris DL, Sheng Y, Zhang Y, Wang YF, Zhu Z, Tombleson P, et al Genome-wide association meta-analysis in Chinese and European individuals identifies ten new loci associated with systemic lupus erythematosus. Nat Genet. 2016;48(8):940–6. doi: 10.1038/ng.3603 27399966 10.1038/ng.3603 PMC4966635

[7] Chung SA, Brown EE, Williams AH, Ramos PS, Berthier CC, Bhangale T, et al Lupus nephritis susceptibility loci in women with systemic lupus erythematosus. J Am Soc Nephrol. 2014;25(12):2859–70. doi: 10.1681/ASN.2013050446 24925725 10.1681/ASN.2013050446 PMC4243339

[8] Freedman BI, Langefeld CD, Andringa KK, Croker Ja, Williams AH, Garner NE, et al End-stage renal disease in African Americans with lupus nephritis is associated with APOL1. Arthritis & rheumatology (Hoboken, NJ). 2014;66(2):390–6. 10.1002/art.38220 PMC4002759 24504811

[9] Chung SA, Tian C, Taylor KE, Lee AT, Ortmann WA, Hom G, et al European population substructure is associated with mucocutaneous manifestations and autoantibody production in systemic lupus erythematosus. Arthritis and Rheumatism. 2009;60(8):2448–56. doi: 10.1002/art.24707 19644962 10.1002/art.24707 PMC2739103

[10] Richman IB, Chung SA, Taylor KE, Kosoy R, Tian C, Ortmann WA, et al European population substructure correlates with systemic lupus erythematosus endophenotypes in North Americans of European descent. Genes Immun. 2010;11(6):515–21. doi: 10.1038/gene.2009.80 19847193 10.1038/gene.2009.80 PMC3951966

[11] Refaat MM, Katz WE. Neoplastic Pericardial Effusion. Clinical Cardiology. 2011;34(10):593–8. doi: 10.1002/clc.20936 21928406 10.1002/clc.20936 PMC6652358

[12] Hoffmann TJ, Zhan Y, Kvale MN, Hesselson SE, Gollub J, Iribarren C, et al Design and coverage of high throughput genotyping arrays optimized for individuals of East Asian, African American, and Latino race/ethnicity using imputation and a novel hybrid SNP selection algorithm. Genomics. 2011;98(6):422–30. doi: 10.1016/j.ygeno.2011.08.007 21903159 10.1016/j.ygeno.2011.08.007 PMC3502750

[13] Das S, Forer L, Schonherr S, Sidore C, Locke AE, Kwong A, et al Next-generation genotype imputation service and methods. Nat Genet. 2016;48(10):1284–7. doi: 10.1038/ng.3656 27571263 10.1038/ng.3656 PMC5157836

[14] Alexander DH, Novembre J, Lange K. Fast model-based estimation of ancestry in unrelated individuals. Genome Res. 2009;19(9):1655–64. doi: 10.1101/gr.094052.109 19648217 10.1101/gr.094052.109 PMC2752134

[15] International HapMap C, Frazer KA, Ballinger DG, Cox DR, Hinds DA, Stuve LL, et al A second generation human haplotype map of over 3.1 million SNPs. Nature. 2007;449(7164):851–61. doi: 10.1038/nature06258 17943122 10.1038/nature06258 PMC2689609

[16] Li JZ, Absher DM, Tang H, Southwick AM, Casto AM, Ramachandran S, et al Worldwide human relationships inferred from genome-wide patterns of variation. Science. 2008;319(5866):1100–4. doi: 10.1126/science.1153717 18292342 10.1126/science.1153717

[17] Huang H, Chanda P, Alonso A, Bader JS, Arking DE. Gene-based tests of association. PLoS Genet. 2011;7(7):e1002177 doi: 10.1371/journal.pgen.1002177 21829371 10.1371/journal.pgen.1002177 PMC3145613

[18] Chang CC, Chow CC, Tellier LC, Vattikuti S, Purcell SM, Lee JJ. Second-generation PLINK: rising to the challenge of larger and richer datasets. Gigascience. 2015;4:7 doi: 10.1186/s13742-015-0047-8 25722852 10.1186/s13742-015-0047-8 PMC4342193

[19] Mishra A, Macgregor S. VEGAS2: Software for More Flexible Gene-Based Testing. Twin Res Hum Genet. 2015;18(1):86–91. doi: 10.1017/thg.2014.79 25518859 10.1017/thg.2014.79

[20] Mishra A, MacGregor S. A Novel Approach for Pathway Analysis of GWAS Data Highlights Role of BMP Signaling and Muscle Cell Differentiation in Colorectal Cancer Susceptibility—Erratum. Twin Res Hum Genet. 2017;20(2):186 doi: 10.1017/thg.2017.6 28228184 10.1017/thg.2017.6

[21] Ioannidis JP, Patsopoulos NA, Evangelou E. Heterogeneity in meta-analyses of genome-wide association investigations. PLoS One. 2007;2(9):e841 doi: 10.1371/journal.pone.0000841 17786212 10.1371/journal.pone.0000841 PMC1950790

[22] Ross KA. Coherent somatic mutation in autoimmune disease. PLoS One. 2014;9(7):e101093 doi: 10.1371/journal.pone.0101093 24988487 10.1371/journal.pone.0101093 PMC4079513

[23] Hatakeyama S. TRIM Family Proteins: Roles in Autophagy, Immunity, and Carcinogenesis. Trends Biochem Sci. 2017;42(4):297–311. doi: 10.1016/j.tibs.2017.01.002 28118948 10.1016/j.tibs.2017.01.002

[24] Kaul A, Gordon C, Crow MK, Touma Z, Urowitz MB, van Vollenhoven R, et al Systemic lupus erythematosus. Nat Rev Dis Primers. 2016;2:16039 doi: 10.1038/nrdp.2016.39 27306639 10.1038/nrdp.2016.39

[25] Crow MK. Long interspersed nuclear elements (LINE-1): potential triggers of systemic autoimmune disease. Autoimmunity. 2010;43(1):7–16. doi: 10.3109/08916930903374865 19961365 10.3109/08916930903374865

[26] Jiang MX, Hong X, Liao BB, Shi SZ, Lai XF, Zheng HY, et al Expression profiling of TRIM protein family in THP1-derived macrophages following TLR stimulation. Sci Rep. 2017;7:42781 doi: 10.1038/srep42781 28211536 10.1038/srep42781 PMC5314404

[27] He DD, Lu Y, Gittelman R, Jin Y, Ling F, Joshua A. Positive selection of the TRIM family regulatory region in primate genomes. Proc Biol Sci. 2016;283(1840). 10.1098/rspb.2016.1602 PMC5069514 27733547

[28] Backes C, Ludwig N, Leidinger P, Harz C, Hoffmann J, Keller A, et al Immunogenicity of autoantigens. BMC Genomics. 2011;12:340 doi: 10.1186/1471-2164-12-340 21726451 10.1186/1471-2164-12-340 PMC3149588

[29] Dall'Era M. Treatment of lupus nephritis: current paradigms and emerging strategies. Curr Opin Rheumatol. 2017;29(3):241–7. doi: 10.1097/BOR.0000000000000381 28207493 10.1097/BOR.0000000000000381

[30] Perl A. Activation of mTOR (mechanistic target of rapamycin) in rheumatic diseases. Nat Rev Rheumatol. 2016;12(3):169–82. doi: 10.1038/nrrheum.2015.172 26698023 10.1038/nrrheum.2015.172 PMC5314913

[31] Stylianou K, Petrakis I, Mavroeidi V, Stratakis S, Vardaki E, Perakis K, et al The PI3K/Akt/mTOR pathway is activated in murine lupus nephritis and downregulated by rapamycin. Nephrol Dial Transplant. 2011;26(2):498–508. doi: 10.1093/ndt/gfq496 20709738 10.1093/ndt/gfq496

[32] Ling F, Kang B, Sun XH. Id proteins: small molecules, mighty regulators. Curr Top Dev Biol. 2014;110:189–216. doi: 10.1016/B978-0-12-405943-6.00005-1 25248477 10.1016/B978-0-12-405943-6.00005-1

[33] Zhao K, He J, Zhang Y, Xu Z, Xiong H, Gong R, et al Activation of FXR protects against renal fibrosis via suppressing Smad3 expression. Sci Rep. 2016;6:37234 doi: 10.1038/srep37234 27853248 10.1038/srep37234 PMC5112546

[34] Zhang Y, Wang S, Liu S, Li C, Wang J. Role of Smad signaling in kidney disease. Int Urol Nephrol. 2015;47(12):1965–75. doi: 10.1007/s11255-015-1115-9 26433882 10.1007/s11255-015-1115-9

[35] Klein-Hessling S, Muhammad K, Klein M, Pusch T, Rudolf R, Floter J, et al NFATc1 controls the cytotoxicity of CD8+ T cells. Nat Commun. 2017;8(1):511 doi: 10.1038/s41467-017-00612-6 28894104 10.1038/s41467-017-00612-6 PMC5593830

[36] Mahajan A, Rodan AR, Le TH, Gaulton KJ, Haessler J, Stilp AM, et al Trans-ethnic Fine Mapping Highlights Kidney-Function Genes Linked to Salt Sensitivity. Am J Hum Genet. 2016;99(3):636–46. doi: 10.1016/j.ajhg.2016.07.012 27588450 10.1016/j.ajhg.2016.07.012 PMC5011075

[37] Miyazaki M, Fujikawa Y, Takita C, Tsumura H. Tacrolimus and cyclosporine A inhibit human osteoclast formation via targeting the calcineurin-dependent NFAT pathway and an activation pathway for c-Jun or MITF in rheumatoid arthritis. Clin Rheumatol. 2007;26(2):231–9. doi: 10.1007/s10067-006-0287-1 16586042 10.1007/s10067-006-0287-1

[38] Keyser P, Borge-Renberg K, Hultmark D. The Drosophila NFAT homolog is involved in salt stress tolerance. Insect Biochem Mol Biol. 2007;37(4):356–62. doi: 10.1016/j.ibmb.2006.12.009 17368199 10.1016/j.ibmb.2006.12.009

[39] Leavy O. T cells: Salt promotes pathogenic TH17 cells. Nat Rev Immunol. 2013;13(4):225 doi: 10.1038/nri3432 23493117 10.1038/nri3432

[40] Wu H, Huang X, Qiu H, Zhao M, Liao W, Yuan S, et al High salt promotes autoimmunity by TET2-induced DNA demethylation and driving the differentiation of Tfh cells. Sci Rep. 2016;6:28065 doi: 10.1038/srep28065 27325182 10.1038/srep28065 PMC4914849

[41] Berry MR, Mathews RJ, Ferdinand JR, Jing C, Loudon KW, Wlodek E, et al Renal Sodium Gradient Orchestrates a Dynamic Antibacterial Defense Zone. Cell. 2017;170(5):860–74 e19. doi: 10.1016/j.cell.2017.07.022 28803730 10.1016/j.cell.2017.07.022

