# Supplementary figures and images for: Genetic contributions to lupus nephritis in a multi-ethnic cohort of systemic lupus erythematous patients
Source: PLoS One. 2018 Jun 28;13(6):e0199003. doi: 10.1371/journal.pone.0199003 (PMC6023154; doi:10.1371/journal.pone.0199003)

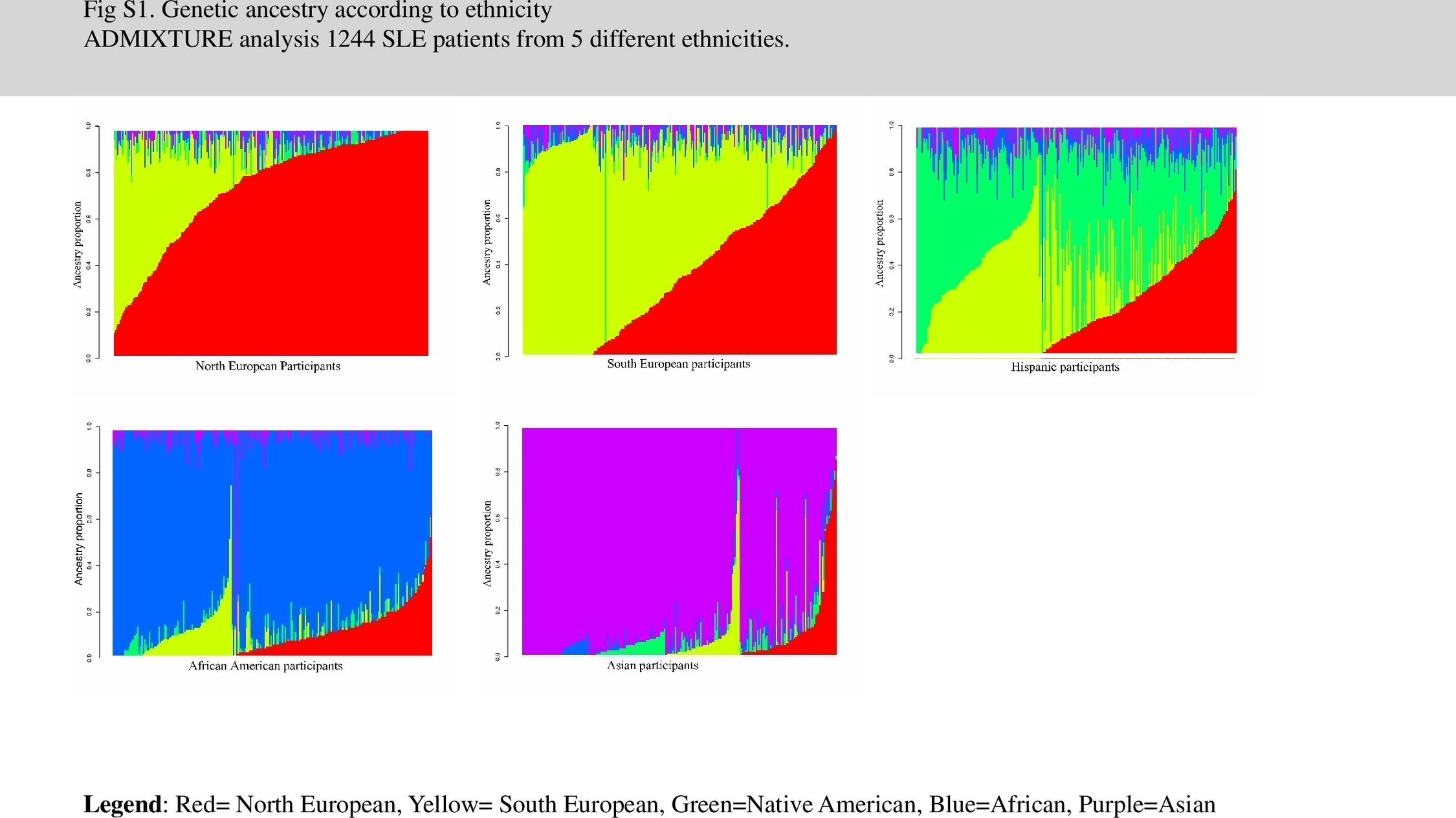

Supplement: S1 Fig — Admixture analysis of 1244 patients with systemic lupus erythematosus from five different ethnicities. (TIF) [file pone.0199003.s001.tif]
