# Supplementary material for: Genetic contributions to lupus nephritis in a multi-ethnic cohort of systemic lupus erythematous patients
Source: PLoS One. 2018 Jun 28;13(6):e0199003. doi: 10.1371/journal.pone.0199003 (PMC6023154; doi:10.1371/journal.pone.0199003)
Supplement: S1 Table — (DOCX) [file pone.0199003.s002.docx]

Supplementary table 1. Association of previously identified loci with estimated glomerular filtration rate (eGFR)

| Chr | Position | Gene | SNP | REF |
| --- | --- | --- | --- | --- |
| 1 | 110014170 | SYPL2 | rs12136063 | ^1^ |
| 1 | 150951477 | LASS2 | rs267734 | ^1; 2^ |
| 1 | 15832281 | CASP9 | rs1800615 | ^1; 2^ |
| 1 | 201016296 | CACNA1S | rs3850625 | ^1^ |
| 1 | 243501763 | SDCCAG8 | rs2802729 | ^1^ |
| 2 | 15793014 | DDX1 | rs807601 | ^1; 2^ |
| 2 | 170008506 | LRP2 | rs4667594 | ^1^ |
| 2 | 211540507 | CPS1 | rs7422339 | ^1; 3^ |
| 2 | 217682779 | IGFBP5 | rs2712184 | ^1^ |
| 2 | 27730940 | GCKR | rs1260326 | ^1; 2^ |
| 2 | 73679280 | ALMS1 | rs6546838 | ^1; 2^ |
| 3 | 13906850 | WNT7A | rs6795744 | ^1^ |
| 3 | 141724644 | TFDP2 | rs2861422 | ^1; 2^ |
| 3 | 170091902 | SKIL | rs9682041 | ^1^ |
| 3 | 185822353 | ETV5 | rs10513801 | ^1^ |
| 4 | 103561709 | NFKB1 | rs228611 | ^1^ |
| 4 | 77368847 | SHROOM3 | rs17319721 | ^1; 3^ |
| 5 | 176817636 | SLC34A1 | rs6420094 | ^1; 2^ |
| 5 | 39397132 | DAB2 | rs11959928 | ^1; 2^ |
| 6 | 160675764 | SLC22A2 | rs316009 | ^1; 2^ |
| 6 | 27341409 | ZNF204 | rs7759001 | ^1^ |
| 6 | 43809802 | VEGFA | rs9472135 | ^1; 2^ |
| 7 | 1285195 | UNCX | rs10277115 | ^1^ |
| 7 | 151407801 | PRKAG2 | rs7805747 | ^1^ |
| 7 | 32919927 | KBTBD2 | rs3750082 | ^1^ |
| 7 | 77555005 | TMEM60 | rs848490 | ^1; 2^ |
| 8 | 23714992 | STC1 | rs3758086 | ^1; 3^ |
| 9 | 71434707 | PIP5K1B | rs4744712 | ^1; 2^ |
| 10 | 1065710 | WDR37 | rs1044261 | ^1; 2^ |
| 10 | 52645424 | A1CF | rs10994860 | ^1^ |
| 11 | 2789955 | KCNQ1 | rs163160 | ^1^ |
| 11 | 30749090 | MPPED2 | rs963837 | ^1; 2^ |
| 11 | 65506822 | AP5B1 | rs4014195 | ^1^ |
| 12 | 15321194 | PTPRO | rs7956634 | ^1^ |
| 12 | 3368093 | TSPAN9 | rs10491967 | ^1^ |
| 12 | 349298 | SLC6A13 | rs10774021 | ^1; 2^ |
| 12 | 57809456 | INHBC | rs1106766 | ^1^ |
| 13 | 72347448 | DACH1 | rs716877 | ^1; 2^ |
| 15 | 41392134 | INO80 | rs476633 | ^1; 2^ |
| 15 | 45698793 | GATM | rs2467853 | ^1; 3^ |
| 15 | 53946593 | WDR72 | rs491567 | ^1; 2^ |
| 15 | 76158983 | UBE2Q2 | rs1394125 | ^1; 2^ |
| 16 | 20366507 | UMOD | rs13329952 | ^1; 3^ |
| 16 | 89708292 | DPEP1 | rs164748 | ^1^ |

Supplementary table 1 continued

| Chr | Position | Gene | SNP | REF |
| --- | --- | --- | --- | --- |
| 17 | 19438321 | SLC47A1 | rs2453580 | ^1; 3^ |
| 17 | 37499949 | CDK12 / FBXL20 | rs9916302 | ^1; 4; 5^ |
| 17 | 59450105 | BCAS3 | rs11657044 | ^1; 2^ |
| 18 | 77164243 | NFATC1 | rs8091180 | ^1; 6^ |
| 19 | 33356891 | SLC7A9 | rs12460876 | ^1; 2^ |
| 19 | 38464262 | SIPA1L3 | rs11666497 | ^1^ |
| 20 | 33285053 | TP53INP2 | rs6088580 | ^1^ |
| 20 | 52732362 | BCAS1 | rs17216707 | ^1^ |

* Beta is the effect on log(eGFRcrea in ml/min/1.73 m2)

References:

1. Pattaro, C., Teumer, A., Gorski, M., Chu, A.Y., Li, M., Mijatovic, V., Garnaas, M., Tin, A., Sorice, R., Li, Y., et al. (2016). Genetic associations at 53 loci highlight cell types and biological pathways relevant for kidney function. Nat Commun 7, 10023.

2. Köttgen, A., Pattaro, C., Böger, C.A., Fuchsberger, C., Olden, M., Glazer, N.L., Parsa, A., Gao, X., Yang, Q., Smith, A.V., et al. (2010). New loci associated with kidney function and chronic kidney disease. Nature genetics 42, 376-384.

3. Kottgen, A., Glazer, N.L., Dehghan, A., Hwang, S.J., Katz, R., Li, M., Yang, Q., Gudnason, V., Launer, L.J., Harris, T.B., et al. (2009). Multiple loci associated with indices of renal function and chronic kidney disease. Nat Genet 41, 712-717.

4. Chasman, D.I., Fuchsberger, C., Pattaro, C., Teumer, A., Boger, C.A., Endlich, K., Olden, M., Chen, M.H., Tin, A., Taliun, D., et al. (2012). Integration of genome-wide association studies with biological knowledge identifies six novel genes related to kidney function. Hum Mol Genet 21, 5329-5343.

5. Pattaro, C., Kottgen, A., Teumer, A., Garnaas, M., Boger, C.A., Fuchsberger, C., Olden, M., Chen, M.H., Tin, A., Taliun, D., et al. (2012). Genome-wide association and functional follow-up reveals new loci for kidney function. PLoS Genet 8, e1002584.

6. Mahajan, A., Rodan, A.R., Le, T.H., Gaulton, K.J., Haessler, J., Stilp, A.M., Kamatani, Y., Zhu, G., Sofer, T., Puri, S., et al. (2016). Trans-ethnic Fine Mapping Highlights Kidney-Function Genes Linked to Salt Sensitivity. Am J Hum Genet 99, 636-646.
