# Supplementary material for: Genetic contributions to lupus nephritis in a multi-ethnic cohort of systemic lupus erythematous patients
Source: PLoS One. 2018 Jun 28;13(6):e0199003. doi: 10.1371/journal.pone.0199003 (PMC6023154; doi:10.1371/journal.pone.0199003)
Supplement: S2 Table — (DOCX) [file pone.0199003.s003.docx]

Supplementary table 2. Association of previously identified loci with lupus nephritis

| Chr | Position | Gene | SNP | REF |
| --- | --- | --- | --- | --- |
| 1 | 197306099 | CRB1, ZBTB41, ASPM, F13B, CFHR5 | rs2786111 | (1) |
| 1 | 42093015 | HIVEP3 | rs752010 | (1) |
| 4 | 101295077 | EMCN | rs2163882 | (1) |
| 4 | 55011769 | PDGFRA, GSX2 | rs1364989 | (1) |
| 4 | 101296603 | EMCN | rs2099365 | (1) |
| 5 | 57214817 | unknown | rs10041935 | (1) |
| 6 | 19823238 | unknown | rs7773456 | (1) |
| 6 | 31170528 | C6orf15-HCG22 | rs9263871 | (1) |
| 6 | 32217232 | C6orf10, NOTCh4 | rs9267972 | (1) |
| 8 | 122275906 | unknown | rs7834765 | (1) |
| 9 | 29632142 | unknown | rs601162 | (1) |
| 10 | 13645210 | PRPF18, FRMD4A | rs2399985 | (1) |
| 12 | 96261568 | FLJ40089 | rs6538678 | (1) |
| 13 | 110856153 | COL4A1 | rs648705 | (1) |
| 14 | 51234160 | NIN, SAV1, SPG3A, MAP4K5 | rs8012283 | (1) |
| 14 | 58559241 | C14orf37 | rs4901847 | (1) |
| 16 | 24898972 | SLC5A11 | rs274068 | (1) |
| 20 | 21142813 | C20orf19, XRN2, NKX2-4, NKX2-2 | rs2236178 | (1) |
| 22 | 36661906 | APOL1 | rs73885319 | (2) |
| 22 | 36662034 | APOL1 | rs60910145 | (2) |

References:

1. Chung SA, Brown EE, Williams AH, Ramos PS, Berthier CC, Bhangale T, et al. Lupus Nephritis Susceptibility Loci in Women with Systemic Lupus Erythematosus. Journal of the American Society of Nephrology : JASN. 2014:ASN.2013050446-ASN.

2. Freedman BI, Langefeld CD, Andringa KK, Croker Ja, Williams AH, Garner NE, et al. End-stage renal disease in African Americans with lupus nephritis is associated with APOL1. Arthritis & rheumatology (Hoboken, NJ). 2014;66(2):390-6.
