# Supplementary material for: Genetic contributions to lupus nephritis in a multi-ethnic cohort of systemic lupus erythematous patients
Source: PLoS One. 2018 Jun 28;13(6):e0199003. doi: 10.1371/journal.pone.0199003 (PMC6023154; doi:10.1371/journal.pone.0199003)
Supplement: S3 Table — (DOCX) [file pone.0199003.s004.docx]

Supplementary Table 3. Association of previously identified loci with systemic lupus erythematosus

| Chr | Position | gene | snp | Association with LN | REF |
| --- | --- | --- | --- | --- | --- |
| 1 | 114377568 | PTPN22 | rs2476601 |  | ^1; 2^ |
| 1 | 161479745 | FCGR2A | rs1801274 | Yes | ^2; 3^ |
| 1 | 161514542 | FCGR3A | rs396991 | Yes | ^4; 5^ |
| 1 | 161643798 | FCGR2B | rs1050501 | Yes | ^5; 6^ |
| 1 | 173164787 | TNFSF4 | rs4916313 |  | ^7^ |
| 1 | 173191475 | TNFSF4 | rs2205960 | Yes | ^8; 9^ |
| 1 | 173307901 | LOC100506023 | rs12736195 |  | ^10^ |
| 1 | 173315625 | TNFSF4 | rs17346550 | Yes | ^10^ |
| 1 | 174312813 | RABGAP1L | rs17301013 |  | ^10^ |
| 1 | 183310860 | NMNAT2-SMG7 | rs111487113 |  | ^7^ |
| 1 | 183353853 | NMNAT2 | rs2022013 |  | ^11^ |
| 1 | 183532437 | NCF2 | rs13306575 |  | ^8; 10^ |
| 1 | 183532580 | NCF2 | rs17849502 |  | ^8; 12^ |
| 1 | 183549757 | NMNAT2 | rs10911363 |  | ^8^ |
| 1 | 206939904 | IL10 | rs3024505 |  | ^1^ |
| 1 | 206955041 | IL10 | rs3122605 |  | ^8^ |
| 1 | 236034274 | LYST | rs2891663 |  | ^1^ |
| 1 | 236039877 | LYST | rs9782955 |  | ^1^ |
| 1 | 67806432 | IL12RB2 | rs1874791 |  | ^1^ |
| 2 | 163124051 | IFIH1 | rs1990760 |  | ^8; 13^ |
| 2 | 163137983 | IFIH1 | rs10930046 |  | ^14^ |
| 2 | 191943742 | STAT4 | rs11889341 | Yes | ^8; 10^ |
| 2 | 191953998 | STAT4 | rs12612769 | Yes | ^7^ |
| 2 | 191958656 | STAT4 | rs4274624 | Yes | ^9^ |
| 2 | 33701890 | RASGRP3 | rs13385731 |  | ^9^ |
| 2 | 61068167 | PAPOLG-LINC01185 | rs1432296 |  | ^8^ |
| 3 | 58321707 | PXK | rs6445972 |  | ^15^ |
| 3 | 58370177 | PXK | rs6445975 |  | ^2; 8^ |
| 4 | 102725298 | BANK1 | rs4699260 |  | ^7^ |
| 4 | 102750922 | BANK1 | rs17266594 |  | ^16^ |
| 4 | 102751076 | BANK1 | rs10516487 |  | ^16; 17^ |
| 4 | 123391464 | IL2-IL21 | rs11724582 |  | ^8^ |
| 4 | 954247 | DGKQ | rs3733345 |  | ^8^ |
| 5 | 100135865 | ST8SIA4 | rs6886392 |  | ^8^ |
| 5 | 133552454 | MGC13017 | rs7704116 |  | ^18^ |
| 5 | 150425032 | TNIP1 | rs62382335 | Yes | ^7^ |
| 5 | 150457485 | TN1P1 | rs7708392 | Yes | ^8; 10^ |
| 5 | 150458146 | TNIP1 | rs10036748 | Yes | ^2; 8^ |
| 5 | 159879978 | miR-146a) | rs2431697 |  | ^19^ |
| 5 | 159886620 | SLU7 | rs2431099 | Yes | ^20^ |
| 5 | 159887336 | PTTG1-MIR146A | rs2431098 |  | ^8^ |
| 6 | 32421227 | unknown | rs116727542 |  | ^21^ |

Supplementary Table 3 continued

| Chr | Position | gene | snp | Association with LN | REF |
| --- | --- | --- | --- | --- | --- |
| 6 | 106588806 | ATG5/PRDM1 | rs6568431 |  | ^1; 9^ |
| 6 | 137973068 | TNFAIP3 | rs2327832 | Yes | ^9^ |
| 6 | 138006504 | TNFAIP3 | rs6920220 | Yes | ^22^ |
| 6 | 138190529 | TNFAIP3 | rs57087937 | Yes | ^7^ |
| 6 | 138289848 | unknown | rs9373203 |  | ^21^ |
| 6 | 31721033 | MSH5 | rs3131379 |  | ^23^ |
| 6 | 32413051 | HLA-DRA | rs3135388 | Yes | ^1; 2; 24^ |
| 6 | 32591198 | HLADXL9 | rs9271593 | Yes | ^7^ |
| 6 | 32605884 | HLA-DQA1 | rs2187668 | Yes | ^10; 32^ |
| 6 | 32626565 | unknown | rs9273371 |  | ^21^ |
| 6 | 32678999 | DQA2-DQB1 | rs9275572 |  | ^10^ |
| 6 | 34812701 | UHRF1BP1-DEF6 | rs34840245 |  | ^8^ |
| 6 | 34824636 | UHRF1BP1 | rs11755393 |  | ^1^ |
| 7 | 128596805 | IRF5/TNP03 | rs12539741 |  | ^10^ |
| 7 | 28167391 | JAZF1 | rs10254284 |  | ^1; 10^ |
| 7 | 28189411 | JAZF1 | rs1635852 |  | ^1^ |
| 7 | 128573967 | IRF5 | rs4728142 | Yes | ^8; 10^ |
| 7 | 128585616 | IRF5-TNPO3 | rs35000415 |  | ^8^ |
| 7 | 128594183 | TNP03 | rs10488631 |  | ^10^ |
| 7 | 128718708 | IRF5_TNPo3 | rs34350562 |  | ^7^ |
| 7 | 28185891 | JAZF1 | rs849142 |  | ^1^ |
| 7 | 50305863 | IKZF1 | rs4917014 | Yes | ^25^ |
| 7 | 75173180 | HIP1 | rs1167796 |  | ^26^ |
| 7 | 8155157 | ICA1 | rs74787882 |  | ^7^ |
| 7 | 8187094 | ICA1 | rs10156091 |  | ^2^ |
| 8 | 11030935 | XKR6 | rs7000132 |  | ^7^ |
| 8 | 11344127 | BLK | rs2618473 | Yes | ^9^ |
| 8 | 11349147 | BLK | rs7822109 | Yes | ^7^ |
| 8 | 11351912 | BLK | rs922483 | Yes | ^27^ |
| 8 | 56849386 | LYN | rs7829816 |  | ^2^ |
| 8 | 56980803 | LYN-RPS20 | rs2953898 |  | ^8^ |
| 8 | 79556148 | PKIA-ZC2HC1A | rs4739134 |  | ^8^ |
| 9 | 102337331 | AK057451 | rs11788118 |  | ^8^ |
| 9 | 34710338 | CCL21 | rs11574914 |  | ^28^ |
| 9 | 7071706 | KDM4C | rs4641121 |  | ^10^ |
| 10 | 105003721 | RPEL1 | rs4917385 |  | ^10^ |
| 10 | 105154089 | USMG5 | rs7911488 |  | ^10^ |
| 10 | 50119054 | WDFY4 | rs1913517 |  | ^9^ |
| 10 | 52957721 | PRKG1 | rs7897633 |  | ^29^ |
| 11 | 128328959 | ETS1 | rs1128334 |  | ^9^ |
| 11 | 118610463 | DDX6 | rs4499035 |  | ^9^ |
| 11 | 118692727 | DDX6 | rs503425 |  | ^1^ |
| 11 | 128310346 | ETS1 | rs34516251 |  | ^7^ |

Supplementary table 3 Continued

| Chr | Position | gene | snp | Association with LN | REF |
| --- | --- | --- | --- | --- | --- |
| 11 | 128311059 | ETS1 | rs6590330 |  | ^7^ |
| 11 | 34780936 | EHF | rs10466455 |  | ^30^ |
| 11 | 35084592 | CD44/PDHX | rs2732552 |  | ^31^ |
| 11 | 35098193 | CD44 | rs387619 |  | ^31^ |
| 11 | 35119482 | PDHX-CD44 | rs353592 |  | ^8^ |
| 11 | 589564 | IRF7 | rs4963128 |  | ^24^ |
| 11 | 613208 | IRF7 | rs1131665 |  | ^24^ |
| 11 | 64354795 | SLC22A12 | rs11231824 |  | ^10^ |
| 11 | 72533536 | FCHSD2 and P2RY2 | rs11235604 |  | ^21^ |
| 11 | 72863697 | FCHSD2/P2RY2 | rs11235667 |  | ^21^ |
| 12 | 112486818 | TRAFD1 | rs17696736 |  | ^32^ |
| 12 | 129275027 | SLC15A4 | rs6486730 |  | ^1^ |
| 12 | 129278864 | SLC15A4 | rs1059312 |  | ^8^ |
| 12 | 129300694 | SLC15A4 | rs1385374 |  | ^9^ |
| 14 | 20940606 | PNP | rs1049564 |  | ^29^ |
| 15 | 48508400 | SLC12A1 | rs1878186 |  | ^10^ |
| 15 | 75079474 | CSK | rs34933034 |  | ^33^ |
| 16 | 30642867 | PRR14 | rs7197475 |  | ^34^ |
| 16 | 31272353 | ITGAM | rs34572943 | Yes | ^10^ |
| 16 | 31313253 | ITGAM | rs9888739 | Yes | ^7^ |
| 16 | 31335906 | ITGAM-ITGAX | rs41476751 | Yes | ^2^ |
| 16 | 57392241 | PLLP-CCL2 | rs223889 |  | ^8^ |
| 16 | 85972598 | IRF8 | rs11648084 |  | ^25^ |
| 16 | 85972612 | IRF8 | rs11644034 |  | ^35^ |
| 16 | 85992649 | IRF8 | rs12444486 |  | ^13^ |
| 17 | 38010815 | IKZF3 | rs8079075 |  | ^36^ |
| 17 | 38023441 | IRF8 | rs1453560 |  | ^2^ |
| 17 | 73312184 | GRB2 | rs8072449 |  | ^7^ |
| 18 | 67530439 | CD226 | rs727088 |  | ^19^ |
| 19 | 10418251 | RAVER1/ZGLP1 | rs35186095 |  | ^7^ |
| 19 | 10473570 | TYK2 | rs91755 |  | ^13^ |
| 19 | 18517767 | LRRC25-SSPB4 | rs13344313 |  | ^8^ |
| 19 | 55737798 | PTPRH-TMEM86B | rs56154925 |  | ^8^ |
| 20 | 44747947 | NCOA5-CD40 | rs4810485 |  | ^8^ |
| 22 | 21921686 | UBE2L3 | rs140490 |  | ^37^ |
| 22 | 21933780 | UBE2L3 | rs181366 |  | ^7^ |
| 22 | 21939675 | UBE2L3 | rs5754217 |  | ^9^ |
| 22 | 40293463 | ENTHD1-GRAP2 | rs137956 |  | ^8^ |
| 22 | 43609760 | SCUBE1 | rs2071725 |  | ^2^ |

References:

1. Gateva, V., Sandling, J.K., Hom, G., Taylor, K.E., Chung, S.A., Sun, X., Ortmann, W., Kosoy, R., Ferreira, R.C., Nordmark, G., et al. (2009). A large-scale replication study identifies TNIP1, PRDM1, JAZF1, UHRF1BP1 and IL10 as risk loci for systemic lupus erythematosus. Nat Genet 41, 1228-1233.

2. International Consortium for Systemic Lupus Erythematosus, G., Harley, J.B., Alarcon-Riquelme, M.E., Criswell, L.A., Jacob, C.O., Kimberly, R.P., Moser, K.L., Tsao, B.P., Vyse, T.J., Langefeld, C.D., et al. (2008). Genome-wide association scan in women with systemic lupus erythematosus identifies susceptibility variants in ITGAM, PXK, KIAA1542 and other loci. Nat Genet 40, 204-210.

3. Karassa, F.B., Trikalinos, T.A., Ioannidis, J.P., and Fcgamma, R.-S.L.E.M.-A.I. (2002). Role of the Fcgamma receptor IIa polymorphism in susceptibility to systemic lupus erythematosus and lupus nephritis: a meta-analysis. Arthritis Rheum 46, 1563-1571.

4. Deng, Y., and Tsao, B.P. (2014). Advances in lupus genetics and epigenetics. Current Opinion in Rheumatology, 1-1.

5. Sullivan, K.E., Jawad, A.F., Piliero, L.M., Kim, N., Luan, X., Goldman, D., and Petri, M. (2003). Analysis of polymorphisms affecting immune complex handling in systemic lupus erythematosus. Rheumatology (Oxford) 42, 446-452.

6. Deng, Y., and Tsao, B.P. (2010). Genetic susceptibility to systemic lupus erythematosus in the genomic era. Nat Rev Rheumatol 6, 683-692.

7. Raj, P., Rai, E., Song, R., Khan, S., Wakeland, B.E., Viswanathan, K., Arana, C., Liang, C., Zhang, B., Dozmorov, I., et al. (2016). Regulatory polymorphisms modulate the expression of HLA class II molecules and promote autoimmunity. Elife 5.

8. Langefeld, C.D., Ainsworth, H.C., Cunninghame Graham, D.S., Kelly, J.A., Comeau, M.E., Marion, M.C., Howard, T.D., Ramos, P.S., Croker, J.A., Morris, D.L., et al. (2017). Transancestral mapping and genetic load in systemic lupus erythematosus. Nat Commun 8, 16021.

9. Han, J.W., Zheng, H.F., Cui, Y., Sun, L.D., Ye, D.Q., Hu, Z., Xu, J.H., Cai, Z.M., Huang, W., Zhao, G.P., et al. (2009). Genome-wide association study in a Chinese Han population identifies nine new susceptibility loci for systemic lupus erythematosus. Nat Genet 41, 1234-1237.

10. Alarcon-Riquelme, M.E., Ziegler, J.T., Molineros, J., Howard, T.D., Moreno-Estrada, A., Sanchez-Rodriguez, E., Ainsworth, H.C., Ortiz-Tello, P., Comeau, M.E., Rasmussen, A., et al. (2016). Genome-Wide Association Study in an Amerindian Ancestry Population Reveals Novel Systemic Lupus Erythematosus Risk Loci and the Role of European Admixture. Arthritis Rheumatol 68, 932-943.

11. Deng, Y., Zhao, J., Sakurai, D., Sestak, A.L., Osadchiy, V., Langefeld, C.D., Kaufman, K.M., Kelly, J.A., James, J.A., Petri, M.A., et al. (2016). Decreased SMG7 expression associates with lupus-risk variants and elevated antinuclear antibody production. Ann Rheum Dis 75, 2007-2013.

12. Jacob, C.O., Eisenstein, M., Dinauer, M.C., Ming, W., Liu, Q., John, S., Quismorio, F.P., Jr., Reiff, A., Myones, B.L., Kaufman, K.M., et al. (2012). Lupus-associated causal mutation in neutrophil cytosolic factor 2 (NCF2) brings unique insights to the structure and function of NADPH oxidase. Proc Natl Acad Sci U S A 109, E59-67.

13. Cunninghame Graham, D.S., Morris, D.L., Bhangale, T.R., Criswell, L.A., Syvanen, A.C., Ronnblom, L., Behrens, T.W., Graham, R.R., and Vyse, T.J. (2011). Association of NCF2, IKZF1, IRF8, IFIH1, and TYK2 with systemic lupus erythematosus. PLoS Genet 7, e1002341.

14. Molineros, J.E., Maiti, A.K., Sun, C., Looger, L.L., Han, S., Kim-Howard, X., Glenn, S., Adler, A., Kelly, J.A., Niewold, T.B., et al. (2013). Admixture mapping in lupus identifies multiple functional variants within IFIH1 associated with apoptosis, inflammation, and autoantibody production. PLoS Genet 9, e1003222.

15. Vaughn, S.E., Foley, C., Lu, X., Patel, Z.H., Zoller, E.E., Magnusen, A.F., Williams, A.H., Ziegler, J.T., Comeau, M.E., Marion, M.C., et al. (2014). Lupus risk variants in the PXK locus alter B-cell receptor internalization. Front Genet 5, 450.

16. Kozyrev, S.V., Abelson, A.K., Wojcik, J., Zaghlool, A., Linga Reddy, M.V., Sanchez, E., Gunnarsson, I., Svenungsson, E., Sturfelt, G., Jonsen, A., et al. (2008). Functional variants in the B-cell gene BANK1 are associated with systemic lupus erythematosus. Nat Genet 40, 211-216.

17. Fan, Y., Tao, J.H., Zhang, L.P., Li, L.H., and Ye, D.Q. (2011). The association between BANK1 and TNFAIP3 gene polymorphisms and systemic lupus erythematosus: a meta-analysis. Int J Immunogenet 38, 151-159.

18. Tan, W., Sunahori, K., Zhao, J., Deng, Y., Kaufman, K.M., Kelly, J.A., Langefeld, C.D., Williams, A.H., Comeau, M.E., Ziegler, J.T., et al. (2011). Association of PPP2CA polymorphisms with systemic lupus erythematosus susceptibility in multiple ethnic groups. Arthritis Rheum 63, 2755-2763.

19. Lofgren, S.E., Frostegard, J., Truedsson, L., Pons-Estel, B.A., D'Alfonso, S., Witte, T., Lauwerys, B.R., Endreffy, E., Kovacs, L., Vasconcelos, C., et al. (2012). Genetic association of miRNA-146a with systemic lupus erythematosus in Europeans through decreased expression of the gene. Genes Immun 13, 268-274.

20. Chung, S.A., Taylor, K.E., Graham, R.R., Nititham, J., Lee, A.T., Ortmann, W.A., Jacob, C.O., Alarcón-Riquelme, M.E., Tsao, B.P., Harley, J.B., et al. (2011). Differential genetic associations for systemic lupus erythematosus based on anti-dsDNA autoantibody production. PLoS Genetics 7.

21. Lee, H.S., Kim, T., Bang, S.Y., Na, Y.J., Kim, I., Kim, K., Kim, J.H., Chung, Y.J., Shin, H.D., Kang, Y.M., et al. (2014). Ethnic specificity of lupus-associated loci identified in a genome-wide association study in Korean women. Ann Rheum Dis 73, 1240-1245.

22. Graham, R.R., Cotsapas, C., Davies, L., Hackett, R., Lessard, C.J., Leon, J.M., Burtt, N.P., Guiducci, C., Parkin, M., Gates, C., et al. (2008). Genetic variants near TNFAIP3 on 6q23 are associated with systemic lupus erythematosus. Nat Genet 40, 1059-1061.

23. Hughes, T., Adler, A., Kelly, J.A., Kaufman, K.M., Williams, A.H., Langefeld, C.D., Brown, E.E., Alarcon, G.S., Kimberly, R.P., Edberg, J.C., et al. (2012). Evidence for gene-gene epistatic interactions among susceptibility loci for systemic lupus erythematosus. Arthritis Rheum 64, 485-492.

24. Fu, Q., Zhao, J., Qian, X., Wong, J.L., Kaufman, K.M., Yu, C.Y., Hwee Siew, H., Tan Tock Seng Hospital Lupus Study, G., Mok, M.Y., Harley, J.B., et al. (2011). Association of a functional IRF7 variant with systemic lupus erythematosus. Arthritis Rheum 63, 749-754.

25. Lessard, C.J., Adrianto, I., Ice, J.A., Wiley, G.B., Kelly, J.A., Glenn, S.B., Adler, A.J., Li, H., Rasmussen, A., Williams, A.H., et al. (2012). Identification of IRF8, TMEM39A, and IKZF3-ZPBP2 as susceptibility loci for systemic lupus erythematosus in a large-scale multiracial replication study. American journal of human genetics 90, 648-660.

26. Okada, Y., Shimane, K., Kochi, Y., Tahira, T., Suzuki, A., Higasa, K., Takahashi, A., Horita, T., Atsumi, T., Ishii, T., et al. (2012). A genome-wide association study identified AFF1 as a susceptibility locus for systemic lupus eyrthematosus in Japanese. PLoS Genet 8, e1002455.

27. Guthridge, J.M., Lu, R., Sun, H., Sun, C., Wiley, G.B., Dominguez, N., Macwana, S.R., Lessard, C.J., Kim-Howard, X., Cobb, B.L., et al. (2014). Two functional lupus-associated BLK promoter variants control cell-type- and developmental-stage-specific transcription. Am J Hum Genet 94, 586-598.

28. Gianfrancesco, M.A., Balzer, L., Taylor, K.E., Trupin, L., Nititham, J., Seldin, M.F., Singer, A.W., Criswell, L.A., and Barcellos, L.F. (2016). Genetic risk and longitudinal disease activity in systemic lupus erythematosus using targeted maximum likelihood estimation. Genes Immun 17, 358-362.

29. Kariuki, S.N., Ghodke-Puranik, Y., Dorschner, J.M., Chrabot, B.S., Kelly, J.A., Tsao, B.P., Kimberly, R.P., Alarcon-Riquelme, M.E., Jacob, C.O., Criswell, L.A., et al. (2015). Genetic analysis of the pathogenic molecular sub-phenotype interferon-alpha identifies multiple novel loci involved in systemic lupus erythematosus. Genes Immun 16, 15-23.

30. Armstrong, D.L., Zidovetzki, R., n-Riquelme, M.E.A.o., Tsao, B.P., Criswell, L.A., Kimberly, R.P., Harley, J.B., Sivils, K.L., Vyse, T.J., Gaffney, P.M., et al. (2014). GWAS identifies novel SLE susceptibility genes and explains the association of the HLA region. 1-8.

31. Lessard, C.J., Adrianto, I., Kelly, J.A., Kaufman, K.M., Grundahl, K.M., Adler, A., Williams, A.H., Gallant, C.J., Marta, E.A.-R.o.b.o.t.B., Networks, G., et al. (2011). Identification of a systemic lupus erythematosus susceptibility locus at 11p13 between PDHX and CD44 in a multiethnic study. Am J Hum Genet 88, 83-91.

32. Prahalad, S., Hansen, S., Whiting, A., Guthery, S.L., Clifford, B., McNally, B., Zeft, A.S., Bohnsack, J.F., and Jorde, L.B. (2009). Variants in TNFAIP3, STAT4, and C12orf30 loci associated with multiple autoimmune diseases are also associated with juvenile idiopathic arthritis. Arthritis Rheum 60, 2124-2130.

33. Manjarrez-Orduno, N., Marasco, E., Chung, S.A., Katz, M.S., Kiridly, J.F., Simpfendorfer, K.R., Freudenberg, J., Ballard, D.H., Nashi, E., Hopkins, T.J., et al. (2012). CSK regulatory polymorphism is associated with systemic lupus erythematosus and influences B-cell signaling and activation. Nat Genet 44, 1227-1230.

34. Wang, C., Ahlford, A., Jarvinen, T.M., Nordmark, G., Eloranta, M.L., Gunnarsson, I., Svenungsson, E., Padyukov, L., Sturfelt, G., Jonsen, A., et al. (2013). Genes identified in Asian SLE GWASs are also associated with SLE in Caucasian populations. Eur J Hum Genet 21, 994-999.

35. Chrabot, B.S., Kariuki, S.N., Zervou, M.I., Feng, X., Arrington, J., Jolly, M., Boumpas, D.T., Reder, A.T., Goulielmos, G.N., and Niewold, T.B. (2013). Genetic variation near IRF8 is associated with serologic and cytokine profiles in systemic lupus erythematosus and multiple sclerosis. Genes Immun 14, 471-478.

36. Lessard, C.J., Adrianto, I., Ice, J.A., Wiley, G.B., Kelly, J.A., Glenn, S.B., Adler, A.J., Li, H., Rasmussen, A., Williams, A.H., et al. (2012). Identification of IRF8, TMEM39A, and IKZF3-ZPBP2 as susceptibility loci for systemic lupus erythematosus in a large-scale multiracial replication study. Am J Hum Genet 90, 648-660.

37. Lewis, M.J., Vyse, S., Shields, A.M., Boeltz, S., Gordon, P.A., Spector, T.D., Lehner, P.J., Walczak, H., and Vyse, T.J. (2015). UBE2L3 polymorphism amplifies NF-kappaB activation and promotes plasma cell development, linking linear ubiquitination to multiple autoimmune diseases. Am J Hum Genet 96, 221-234.
