# Supplementary material for: Genetic contributions to lupus nephritis in a multi-ethnic cohort of systemic lupus erythematous patients
Source: PLoS One. 2018 Jun 28;13(6):e0199003. doi: 10.1371/journal.pone.0199003 (PMC6023154; doi:10.1371/journal.pone.0199003)
Supplement: S4 Table — (DOCX) [file pone.0199003.s005.docx]

| **Supplementary table 4. Genes with the strongest evidence of association with lupus nephritis among South European SLE patients*** | | | | | | | | | |
| --- | --- | --- | --- | --- | --- | --- | --- | --- | --- |
| Chr | Gene | nSNPs | nSims | Start | Stop | Test | Pvalue | TopSNP | TopSNP-pvalue |
| 19 | ZNF546 | 14 | 1.00E+06 | 40452942 | 40573514 | 42.40404 | 1.00E-06 | rs3754 | 0.001777 |
| 6 | TRIM10 | 40 | 1.00E+06 | 30069722 | 30178711 | 36.10366 | 1.00E-06 | rs116227354 | 0.02467 |
| 6 | TRIM15 | 37 | 1.00E+06 | 30080982 | 30190473 | 34.12671 | 1.00E-06 | rs116227354 | 0.02467 |
| 19 | ZNF780B | 20 | 1.00E+06 | 40484166 | 40612115 | 48.00545 | 1.20E-05 | rs3754 | 0.001777 |
| 6 | TRIM26 | 28 | 1.00E+06 | 30102231 | 30231271 | 24.60001 | 2.20E-05 | rs116227354 | 0.02467 |
| 6 | TRIM31 | 38 | 1.00E+06 | 30020673 | 30130867 | 37.38552 | 4.10E-05 | rs116227354 | 0.02467 |
| 6 | TRIM40 | 42 | 1.00E+06 | 30053884 | 30166512 | 36.50064 | 6.00E-05 | rs116227354 | 0.02467 |
| 8 | MIR599 | 6 | 1.00E+06 | 1E+08 | 1.01E+08 | 27.34462 | 0.000328 | rs16897440 | 0.003868 |
| 8 | MIR875 | 6 | 1.00E+06 | 1E+08 | 1.01E+08 | 27.34462 | 0.000355 | rs16897440 | 0.003868 |
| 8 | HOOK3 | 14 | 1.00E+06 | 42702032 | 42935682 | 38.34307 | 0.000504 | rs10106661 | 0.00183 |
| 17 | MIR3186 | 24 | 1.00E+06 | 79368129 | 79468214 | 86.245 | 0.000538 | rs8077665 | 0.000595 |
| 4 | PRSS12 | 28 | 1.00E+06 | 1.19E+08 | 1.19E+08 | 89.24266 | 0.000602 | rs12498329 | 0.000305 |
| 22 | UQCR10 | 13 | 1.00E+06 | 30113357 | 30216402 | 58.14975 | 0.000754 | rs140120 | 0.000119 |
| 16 | MIR7854 | 46 | 1.00E+06 | 81517506 | 81617571 | 179.6517 | 0.000764 | rs2925979 | 0.000181 |
| 19 | PSMC4 | 22 | 1.00E+06 | 40426911 | 40537671 | 62.57891 | 0.000921 | rs3754 | 0.001777 |
| 4 | RASL11B | 16 | 1.00E+06 | 53678494 | 53783002 | 82.79583 | 0.000962 | rs11940896 | 0.000373 |
| 1 | FASLG | 31 | 1.00E+05 | 1.73E+08 | 1.73E+08 | 114.0894 | 0.00104 | rs2859247 | 2.16E-05 |
| 22 | ASCC2 | 17 | 1.00E+05 | 30134596 | 30284293 | 65.07338 | 0.00106 | rs140120 | 0.000119 |
| 9 | OLFM1 | 76 | 1.00E+05 | 1.38E+08 | 1.38E+08 | 214.3829 | 0.0011 | rs61318287 | 2.03E-05 |
| 9 | ENTPD8 | 25 | 1.00E+05 | 1.4E+08 | 1.4E+08 | 84.6066 | 0.00133 | rs7468804 | 0.000523 |
| 11 | LINC00678 | 32 | 1.00E+05 | 27589172 | 27706174 | 144.5441 | 0.00138 | rs11030094 | 0.000591 |
| 8 | CHRNB3 | 27 | 1.00E+05 | 42502561 | 42642209 | 81.68891 | 0.00144 | rs2304297 | 0.001831 |
| 10 | PANK1 | 46 | 1.00E+05 | 91289253 | 91455329 | 113.4015 | 0.00149 | rs7098988 | 0.000406 |
| 1 | TXNDC12 | 11 | 1.00E+05 | 52435803 | 52571843 | 53.5375 | 0.00152 | rs7512076 | 0.00121 |
| 4 | NKX1-1 | 14 | 1.00E+05 | 1346719 | 1450230 | 73.64856 | 0.00152 | rs11731672 | 0.000963 |
| 12 | MRPS35 | 39 | 1.00E+05 | 27813705 | 27959237 | 125.039 | 0.00158 | rs1010096 | 0.001141 |
| 9 | MIR23B | 12 | 1.00E+05 | 97797489 | 97897586 | 41.34188 | 0.00158 | rs4744442 | 0.001339 |
| 17 | BAHCC1 | 39 | 1.00E+05 | 79323520 | 79483358 | 116.8089 | 0.0016 | rs8077665 | 0.000595 |
| 9 | MIR27B | 12 | 1.00E+05 | 97797726 | 97897823 | 41.34188 | 0.00164 | rs4744442 | 0.001339 |
| 9 | NOXA1 | 26 | 1.00E+05 | 1.4E+08 | 1.4E+08 | 86.10345 | 0.0017 | rs7468804 | 0.000523 |

*nSNPs=Number of SNPs, nSims=Number of simulations, Start=Start position, Stop=stop position, Test=Gene-based test statistic
