# Supplementary material for: Genetic contributions to lupus nephritis in a multi-ethnic cohort of systemic lupus erythematous patients
Source: PLoS One. 2018 Jun 28;13(6):e0199003. doi: 10.1371/journal.pone.0199003 (PMC6023154; doi:10.1371/journal.pone.0199003)
Supplement: S5 Table — (DOCX) [file pone.0199003.s006.docx]

| **Supplementary table 5. Associations between lupus nephritis and ethnic-specific genetic risk scores (GRS)** | | | | | | | | | | | | |
| --- | --- | --- | --- | --- | --- | --- | --- | --- | --- | --- | --- | --- |
| GRS* | North European | | South European | | Hispanic | | African American | | Asian | | P value: LN status in all participants | P value: LN status in GRS-derived ethnic group |
|  | LN (+) | LN (-) | LN (+) | LN (-) | LN (+) | LN (-) | LN (+) | LN (-) | LN (+) | LN (-) |  |  |
| NE  mean (+/- SD) | 7.4 (2.1) | 6.3 (2.2) | 7 (2.3) | 6.5 (2.2) | 5 (2.2) | 5.1 (2.2) | 3.7 (1.7) | 3.8 (1.7) | 3.3 (1.5) | 3 (1.3) | **0.0001** | **0.03** |
| SE  mean (+/- SD) | 15.3 (2.8) | 14.8 (2.6) | 15 (2.7) | 15.2 (2.5) | 15.6 (2.4) | 15.8 (2.3) | 14.1 (2.1) | 14 (2) | 14.5(1.7) | 14.5 (1.7) | 0.5 | 0.6 |
| HI  mean (+/- SD) | 15.7 (2.2) | 15.7 (2.1) | 15.4 (2.4) | 15.1 (2.5) | 15.5 (2.1) | 15.5 (2) | 15.8 (1.8) | 15.9 (1.9) | 15.1 (1.8) | 15.2 (1.7) | 0.76 | 0.9 |
| AA  mean (+/- SD) | 11.6 (1.8) | 11.5 (1.8) | 11.5 (1.7) | 11.4 (1.8) | 11.2 (1.7) | 11.5 (1.7) | 11.3 (2) | 11.2 (1.8) | 10.7 (1.7) | 10.6 (1.5) | 0.35 | 0.2 |
| AS  mean (+/- SD) | 8.1 (1.8) | 8 (1.6) | 8.2 (1.7) | 8 (1.6) | 8 (1.5) | 8 (1.6) | 8 (1.6) | 8 (1.5) | 7.2 (1.5) | 7.3 (1.5) | 0.7 | 0.5 |

*genetic risk score (GRS) for each ethnic group, comprised of SNPs with p<0.05 for association with LN in that ethnic group. LN=lupus nephritis
